# Prevalence of Low Back Pain and Dorsalgia and Associated Factors among Casual Dockworkers

**DOI:** 10.3390/ijerph15102310

**Published:** 2018-10-20

**Authors:** Marta Regina Cezar-Vaz, Clarice Alves Bonow, Daiani Modernel Xavier, Joana Cezar Vaz, Letícia Silveira Cardoso, Marlise Capa Verde Almeida de Mello, Valdecir Zavarese da Costa, Cynthia Fontella Sant’Anna

**Affiliations:** 1 School of Nursing, Federal University of Rio Grande, Rio Grande, RS 96203-900, Brazil; daiamoder@gmail.com (D.M.X.); marlisealmeida@msn.com (M.C.V.A.d.M.); 2 Faculty of Nursing, Federal University of Pelotas; Pelotas, RS 96075-630, Brazil; claricebonow@gmail.com; 3 Fundação Getúlio Vargas, Rio de Janeiro, RJ 22250-900, Brazil; joanacezarvaz@yahoo.com.br; 4 Department of Nursing, Federal University of Pampa, Uruguaiana, RS 97501-970, Brazil; lsc_enf@yahoo.com.br (L.S.C.); cynthiafs_enf@yahoo.com.br (C.F.S.); 5 Department of Nursing, Federal University of Santa Maria, Santa Maria, RS 97105-900, Brazil; valdecircosta2005@yahoo.com.br

**Keywords:** musculoskeletal disorders, occupational health, casual dockworkers

## Abstract

This study’s aim was to analyse the relationship between musculoskeletal disorders (low back pain and dorsalgia) and sociodemographic characteristics, workload and occupational hazards among casual dockworkers. This cross-sectional study addressed casual dockworkers from the state of Rio Grande do Sul, Brazil. The convenience sample was composed of 232 casual dockworkers. Data were collected using a structured interview and observation. Poisson regression analysis was used. Association between low back pain and physiological occupational risk (*p* = 0.006), total exertion levels (*p* = 0.014) and frustration (*p* = 0.020) remained statistically significant, while the use of illicit drugs (*p* = 0.023), being a quayside worker (*p* = 0.021) and physiological occupational risk (*p* = 0.040) remained associated with dorsalgia. Decreasing these variables in the workplace may also reduce the prevalence of musculoskeletal disorders such as low back pain and dorsalgia.

## 1. Introduction

The United Nations Conference on Trade and Development [1] estimates cargo transported by sea will be in continuous expansion, with an annual growth of 3.2% between 2017 and 2022 worldwide. Such growth is mainly due to an expansion in the trade of containers and bulk goods. From this perspective, maritime transport will remain the most important means of transportation for the international trade of goods. According to a survey conducted by the CNT (National Confederation of Transportation), in 2011, 95.9% of Brazil’s exports went through seaports [2].

In this context, dockworkers handle and move goods within a port’s premises, on the decks or in the holds of the vessels; receive, check, organize the goods internally, open some for customs conference; handling, stowing and delivering, as well as loading and unloading vessels [3]. This type of work, together with working conditions, directly affects productivity. Such processes, both in Brazil and in other countries around the world, cause transitory or definitive harm to the health conditions of different groups of workers [4,5]. 

Specifically among casual dockworkers, the frequent handling of loads may lead to musculoskeletal disorders (MSDs), which include health problems related to the locomotor system, such as muscles, tendons, skeleton, cartilage, ligaments and nerves [6]. Additionally, a WHO document outlines a goal, to be achieved between 2015 and 2025, of making advancements regarding factors that cause occupational diseases, among which are ergonomic stressors [7] that cause MSDs.

Studies addressing dockworkers report that MSDs frequently affect these workers. One study conducted in the Republic of the Philippines with 290 dockworkers reports that the prevalence and severity of work-related MSDs was significantly greater in the upper and lower back, shoulder and forearm. Additionally, various factors were related to the severity of MSDs, such as age, working time, number of working days per week, level of job satisfaction and perception of safety at work, showing that the aetiology of this condition is multifaceted [8]. In Brazil, one study intended to characterize longshoremen—one type of port worker—shows that one of the main health problems reported by these workers is MSDs, more specifically, lumbar herniated disk and degeneration of the knee joint [9].

MSDs among dockworkers may be associated with the use of work tools such as pliers, for instance, which may lead to clinical manifestations like pain. One study, conducted with construction workers from a shipyard, reports that back pain interfered in the work of 46.5% of the individuals (“occasionally” in 40.4% and “continually” in 6.1%) [10]. Note there are few studies addressing the health of this population of dockworkers, showing the relevance of conducting studies with these workers.

Heavy workload may also account for the prevalence of MSDs among dockworkers. One study addressing the workload of longshoremen shows that their physical workload is quite heavy due to the manual activities these workers must perform, among which is lifting 50 kg sacks of grain. In addition to physical strain, mental demands are also high because dockworkers must pay attention and concentrate on the job [11]. Studies assessing workload among workers performing different functions, such as the study addressing 194 male workers from a textile mill in Iran, report that age affects an individual’s performance, that is, his/her ability to perform certain tasks declines with age [12]. From this perspective, the development of MSDs also advances with age, especially low back pain. It is estimated that the prevalence of MSDs worldwide is between 1.0% and 58.1%, being aggravated from the third decade on and declining at the age of 60 years old [13]. Increased prevalence around the age of 30 is related to labour because this is an age group when people are productive.

Another aspect addressed in this study is the perception of dockworkers in regard to the risk to which they are exposed at work. Risk perception is a decision-making process that takes place when in the face of external conditions presented in daily life that threaten the health and safety of people [14,15,16,17], which is a topic of global interest. In this study, we are interested in acquiring greater understanding of occupational risk perception. In this context, it is a concept directly linked to the different safety behaviour sin the workplace [18]. In other words, there are in the same workplace different risks and different factors that change the perception of workers regarding the risks to which they are expose. From this perspective, the question we want to answer is—What factors do dockworkers perceive as related to manifestations of the musculoskeletal system (low back pain and dorsalgia)?

Therefore, this study’s objective was to analyse the relationship between musculoskeletal system disorders (dorsalgia and low back pain), sociodemographic characteristics, workload and occupational risks of dockworkers.

## 2. Materials and Methods 

Cross-sectional study conducted with dockworkers from the state of Rio Grande do Sul, Brazil. The convenience sample was composed of 232 causal dockworkers out of a total of 579 workers. The sample size was calculated using the EPI INFO version 7.2 and was based on the study of Seadilla and Matias [19]. The significance level was set at 5%, power at 95% and the prevalence of dorsalgia and low back pain was estimated at 50%, with a prevalence ratio estimated at 2. A minimum of 231 subjects was obtained, out of a total of 579.

This study sample was composed of quayside workers, longshoremen and cargo clerks. The work of quayside workers consists of moving cargo within the port, while longshoremen are responsible for moving cargo on the decks or in the holds of the vessels and cargo checkers work inside the vessels and inside the port, checking goods [20]. 

Data were collected through structured interviews and observation of workers from January to December 2014. The questionnaire—a structured interview—was applied at the participants’ workplace at the time they arrived for work in the port. Data collection was organized in order to include all the shifts for which workers are called. Dockworkers have a six-hour daily work journey with four shifts. There are calls every day (from Sunday to Monday): at 6:30 a.m. for the 7 a.m. to 1 p.m. shift; at 12:30 p.m. for the 1 p.m. to 7 p.m. shift; at 6:30 p.m. for the 7 p.m. to 1 a.m. shift; and for the 1 a.m. to 7 a.m. shift. The idea to apply the questionnaire in this place was strategic to enable all dockworkers to participate in the study. All workers were invited regardless of having MSD or not.

The questionnaire addressed sociodemographic variables (age, race, marital status and education); variables regarding the use of legal drugs (tobacco, alcohol and self-medication) and illegal drugs (marijuana and cocaine); occupational variables (function, time working in the function, monthly income, daily work hours, work shifts, workload and exposure to physical, chemical, biological, physiological and/or psychosocial occupational hazards); and variables concerning MSD (dorsalgia and low back pain) and complaints of pain, cramps, or numbness in anatomical regions such as the neck, upper back, middle back and lower back. A numerical scale from 0 to 10 was used to identify level of discomfort. Scores ≤5 indicate mild discomfort, 6 to 7 indicate moderate discomfort and ≥8 indicate severe discomfort [21]. Specifically, questions addressing illegal drugs included whether dockworkers used illegal drugs at work or were aware their colleagues used illegal drugs at work—amphetamines, cannabis, cocaine, heroine or ecstasy. These variables are better explored in an intervention study addressing the use of illegal drugs and infectious-contagious diseases among dockworkers [22].

The variables of the structured questionnaire (socio-demographic, use of legal and illegal drugs, occupational and concerning MSD) were tested and suitable as a set in meetings of the research group and through a pilot study in time prior to data collection, with a sample of ten subjects in different port categories. The main propositions of this preliminary study were designed to assess and enhance the use of the data collection instrument, in regard to its efficacy in the application and cognitive apprehension of the participants its easiness or difficulty in response to the request, just as enhancing qualification of field researchers.

The International Diseases Classification-ICD-10 [23] was used to classify dorsalgia and low back pain. Low back pain is classified as loin pain, low back strain or lumbago not otherwise specified (M54.5 low back pain), while dorsalgia is classified as backache not otherwise specified (M54.9 dorsalgia, unspecified). The latter was reported by the dockworkers, a complaint that was accompanied by symptoms at the time of data collection (back pain in the thoracic region—dorsalgia and lumbar region—low back pain) and also by prior medical diagnosis.

The NASA-TLX questionnaire (National Aeronautics and Space Administration Task Load Index) [24] was used to assess workload, a variable included in the occupational variables. The NASA-TLX is a scale validated, that measures workload through six scales: mental (thinking, choosing, calculating and decision-making); physical (pulling, pushing, shifting items and lifting weight); temporal (amount of time necessary to perform tasks); performance (quality and agility with which tasks are performed), total effort (physical and mental requirements to perform tasks); and frustration (motivation, satisfaction, discouragement and irritation regarding tasks) [25]. Dockworkers rated from 1 to 20 the workload of each demand; higher scores indicate heavier workload. 

The Occupational Health and Safety Act [26], which classifies occupational risks as physical, chemical, biological, physiological or psychosocial, was used to analyse exposure to workplace hazards. Physical risks include noise, vibration, ionizing and non-ionizing radiation (ultraviolet and infrared radiation) and electromagnetic fields. Chemical risks include exposure to harmful chemical products such as lead, mercury, benzene, asbestos, among others and materials containing such chemicals. Biological risks are microorganisms such as bacteria, viruses and fungi. Physiological risks include heavy physical exertion, repetitive movements of the same type, physical positions and working postures that cause fatigue. Psychological risks refer to monotonous work or work that is not appropriate to a worker’s skills, poor organization of work and working alone for long periods of time, for example manual lifting tasks, pushing and pulling, handling low loads at high frequency and working tasks while maintaining fixed postures.

Systematized observation was used to collect data from dockworkers during tasks performed inside the port, focusing on body positions assumed at work. Observation was guided by a checklist based on the characteristics of the port environment and contained information that characterized dockworkers and characteristics of the positions workers adopted during the performance of tasks (sitting, standing, etc.). The workers’ body positions were assessed according to the guidelines provided by the World Health Organization [6]. Direct observation was used without participating in or influencing the dockworkers’ tasks. One pair of observers, members of the Laboratory of Socio-Environmental Studies and Processes and Collective Production of Health, observed one dockworker at a time to better capture the details of the tasks performed, conferring greater reliability and rigor onto data collection.

Observation took place during weekdays for an average of 8 h to 9 h daily, in the morning and afternoon shifts (there was no workplace safety committee available during the evening and night shifts), from July to December 2014. A total of 89 body positions were observed during the work of 66 dockworkers. Workers may have been observed at different points in time performing different tasks; that is, given the characteristic inherent to work in a port, the same worker may perform different tasks in different environments. Workers from three professions were observed: quayside workers, longshoremen and cargo checkers. Observation focused on those workers who present important statistical results in order to identify situations that aggravated dorsalgia and low back pain. All the observed tasks took place on the dock of the port. No tasks were observed in the interior of vessels due to the ship security plan established by the ISPS Code (International Ship and Port Facility Security Code) [27].

In data analysis, the quantitative variables were described by mean and standard deviation or median and interquartile range. Categorical variables were described by absolute and relative frequencies. The Shapiro-Wilk test was applied to assess the normality of continuous data. In order to compare means between dockworkers with and without the presence of the outcome (low back pain or dorsalgia), Student’s *t* test for independent samples was applied. In the case of asymmetry (presence of extreme values), the Mann-Whitney test was used. To compare proportions between dockworkers with and without the presence of the outcome, Pearson’s chi-square or Fisher’s exact tests were used and the Poisson Regression analysis was adopted to control for confounding factors. The criterion used to include variables in the model was presenting *p*-value < 0.20 in the bivariate analysis and the criterion for it to remain in the model was presenting a *p*-value < 0.10 in the final model. The significance level adopted was 5% (*p* ≤ 0.05) and analyses were performed in the SPSS program version 21.0 (IBM, Armonk, NY, USA).

All subjects voluntarily signed free and informed consent forms to be included in the study, which was conducted in accordance with the Declaration of Helsinki and the protocol was approved by the Institutional Review Board at the Federal University of Rio Grande (No. 23116.004481/2013-53).

## 3. Results

The sample was composed of 232 men aged 48.7 years old (±10.4) on average, with most between 40 and 59 years of age (60.3%); were Caucasian (56%); married (60.8%); and alcoholic (51.3%). The sample’s characterization is presented in Table 1.

The predominant number of participants had completed high school (37.1%) and had a median monthly income of R$3800.00 (equivalent to $1022.70); 25.4% were smokers and 12.5% used some type of illegal drug in their daily routines. The drugs most frequently used were marijuana (n = 27; 11.6%) and cocaine (n = 6; 2.6%).

The function that appeared predominant in the work data was that of quayside workers (58.6%). Time working in the function was high, with a mean of 24.3 years (±11.4). Workdays comprised 7.2 h (±2.4) on average. The robust variable (time vs. hours) presented a median of 150 h vs. years and most individuals worked both on the day and the night shifts, depending on what was available on the schedule (76.7%).The scores assigned to the workload demanded by the tasks performed at the port and the work data are presented in Table 2. These rates, ranging from 1 to 20, were assigned by the dockworkers to determine the workload of each task; higher rates indicate heavier workloads.

**Figure 1:**
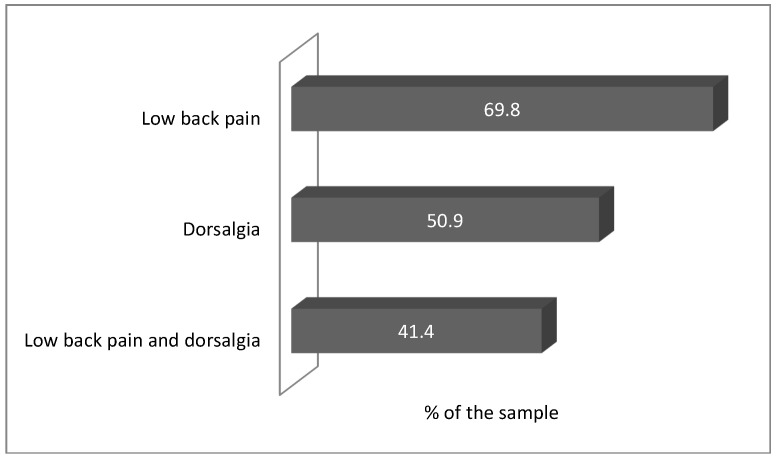
Frequency of dorsalgia and low back pain among dockworkers.

The most frequent musculoskeletal disorders caused by the work performed at the port are presented in Figure 1.

Both outcomes were associated with the study’s remaining variables and these associations are presented in Table 3.

Low back pain appears associated with marital status (*p* = 0.049), working hours (*p* = 0.025), self-medication for musculoskeletal pain (*p* = 0.002) and physical (*p* = 0.008) and temporal (*p* = 0.027) demands, performance (*p* = 0.012), total effort (*p* = 0.001) and frustration (*p* = 0.001). Widowed individuals were less likely to experience low back pain, while workers with a higher average number of working hours, who self-medicated for musculoskeletal pain and with higher levels of workload, were more likely to experience low back pain.

Only self-medication for musculoskeletal pain was significantly associated with dorsalgia (*p* = 0.029), indicating that dockworkers who self-medicated were more likely to experience back pain. No statistically significant association was found between professional function and low back pain (*p* > 0.05), though a significant association was found between being a quayside worker and dorsalgia (*p* = 0.003). Quayside workers presented a higher prevalence of dorsalgia when compared to workers performing other functions (65.3% vs. 44.7%).

The rates assigned by casual dockworkers for pain, cramps and numbness on the neck, upper back, middle back and low back are presented in Table 4.

Table 5 presents the relationship between occupational risks and the outcomes. Workers with low back pain were more frequently exposed to physical (*p* = 0.048), psychosocial (*p* = 0.019) and physiological risks (*p* = 0.001), while dockworkers with dorsalgia were more frequently exposed to chemical (*p* = 0.028), biological (*p* = 0.010) and physiological (*p* = 0.031) occupational risks.

Variables that presented *p* < 0.20 in the bivariate analysis were included in the Poisson multivariate regression model to control for confounding factors, though only variables with *p* <0.10 remained in the final model.

After adjustment, physiological occupational risk (*p* = 0.006), total effort (*p* = 0.014) and frustration (*p* = 0.020) remained statistically associated with low back pain. Individuals at a greater physiological risk present a 32% higher prevalence of low back pain (RP = 1.32; CI 95%: 1.08 to 1.60). Dockworkers with an extra point in the levels of total effort and frustration were 3% and 1% more likely to respectively present low back pain (RP = 1.03; CI 95%: 1.01–1.05 and RP = 1.01; CI 95%: 1.00–1.03), as presented in Table 6.

The following remained associated with dorsalgia: the use of illegal drugs (*p* = 0.023); being a longshoreman (*p* = 0.021); and physiological occupational risk (*p* = 0.040). Dockworkers who reported the use of illegal drugs presented a 34% higher prevalence of dorsalgia (RP = 1.34; CI 95%: 1.04–1.73). Additionally, longshoremen were 39% more likely to present this outcome (RP = 1.39; CI 95%: 1.05–1.85). Finally, workers who experienced greater exposure to physiological occupational risk presented a 30% greater prevalence of dorsalgia (RP = 1.30; CI 95%: 1.01–1.67) when compared to those not exposed to such risk.

In regard to data collected through observation, 89 positions were observed among 66 dockworkers at work; 77 (88.5%) positions were observed among quayside workers, seven (8.1%) among longshoremen and three (3.4%) among cargo checkers. Remaining data are presented in Table 7.

## 4. Discussion

According to the symptoms reported and prior medical diagnoses, most dockworkers presented low back pain (n = 162, 69.8%) and dorsalgia (n = 118, 50.9%). This study does not include analysis of imaging tests, which constitutes a limitation. Self-reports associated with prior medical diagnoses, however, are the first stage to establish a nexus between MSD and labour. Additionally, similar results are found among the same (longshoremen) and different (office workers) populations. One study conducted in the port of the Republic of the Philippines reports a prevalence of 60% of MSD in the upper back and 58% in the low back [8]. Workers with different functions—office workers—present a similar prevalence of MSD in the lower back (49.7%) and cervical (49%) regions [28].

The prevalence of MSD as reported by dockworkers is similar to the prevalence of MSD among other types of workers, which hinders the identification of a causal nexus between health, labour and disease. The literature, however, shows that port work, as presented by one study [29] conducted in two ports in the northeast of Brazil (Mucuripe and Pecém) comparing the tasks and occupational risks to which dockworkers are exposed after changes were implemented by the Port Modernization Act [30]. The authors report that modernization changed the way work is organized and working relations in the two ports are now precarious. The reason is that, despite the technological innovations implemented, the process brought about new occupational hazards and led to the intensification of the work pace, even though work hours were reduced from 12 h to 6 h [29]. Although the study did not compare back pain and low back pain before and after the modernization process took place in the ports, it is interesting to note that workers still experience occupational disorders despite the changes implemented. 

The results from the Poisson regression analysis indicate that workers with higher workload levels for total effort and frustration present a greater prevalence of low back pain; quayside workers using illegal drugs present a greater prevalence of dorsalgia; and workers experiencing greater exposure to physiological occupational risk present a greater prevalence of both dorsalgia and low back pain. Additionally, observation revealed that quayside workers utilize body positions that compromise the musculoskeletal system. Most of these workers were observed in static positions (n = 19, 33.9%) and awkward static positions (n = 34, 60.7%). The World Health Organization recommends dynamic positions over static ones, while the ideal would be a combination of active and inactive periods for relaxation [6]. How many times workers assumed these positions was not counted, which is a limitation of this study. The workers, however, were observed during their working hours and periods of relaxation were not observed. 

The quayside workers were the only ones observed lifting heavy loads. Heavy lifting may cause fatigue and/or overload the musculoskeletal system [6]. One study conducted in Iran with construction workers assuming positions and handling tools reports that these workers lift many heavy loads [31]. Even though construction and port workers (quayside workers) are different professions, their work is similar, as both are required to load and unload materials and frequently change body positions—for instance, getting up and getting down. Thus, ideally, the body’s functional capacity for individuals required to handle heavy loads, as is the case of dockworkers and construction workers, should be assessed [6]. 

Dockworkers with higher scores of workload assigned to total effort (physical and mental requirements to perform tasks) and frustration (motivation, satisfaction, discouragement and irritation toward tasks) [25] are more likely to experience low back pain. One study conducted with different workers who perform a great deal of physical exertion—jute factory workers—demonstrates that occupational strain may lead to MSD [32].

Specifically, quayside workers handle, receive, check and internally move loads, opening volumes for customs conference, handling, stowing, delivering, loading and unloading vessels [3]. Goods include: containers (loaded with clothing, meat, computers); liquid products (fuels and vegetable oils); solid products (grains, coal, cement); fractionated products (paper, wood, steel rolls, wind turbines); and rollon/roll off (cars, buses, trucks, agricultural vehicles and cranes) [33]. The quayside work is a technical job and, in some countries, is performed by robots. The automation of the work performed in ports, however, still requires an individual to handle the robots [34]. This situation is presented in a report addressing the development of activities in the port of Gotemburgo–Sweden [35]. The report shows that, even though advancements are achieved with automation, the work process of dockworkers remains the same because the relationships and risks to which workers are exposed remain the same, despite the reduced number of workers. 

A previous study conducted with the same group of workers [22] shows that quayside workers present a prevalence of 123% greater illegal drug use, a variable that appears in this study as being related to dorsalgia. These results reinforce the idea that the work performed by quayside workers requires considerable physical exertion, which may result in musculoskeletal wear and, thus, cause MDS such as those reported in this study: low back pain and dorsalgia. One of the drugs used by this group of workers as reported in the previously mentioned study [22], cocaine, has an effect on the human brain, euphoria [36], which may serve as ajustification of the use of drugs among workers experiencing intense workload and MSD. In addition to illegal drugs, one study addressing dockworkers from the Republic of the Philippines [8] indicates a relationship between the severity of MSD and the consumption of legal drugs (alcohol and cigarette), which may require further studies to reach a deeper understanding of such relationship.

Physiological occupational risk, a variable related to increased low back pain and dorsalgia experienced by this study’s participants, includes heavy physical exertion, repetitive movements of the same type, physical positions and movements performed at work that cause fatigue [26]. The work performed in ports requires workers to lift heavy objects, hold standing positions and perform repetitive movements, occupational risks that lead to MSD. One study performed in a Brazilian port [9] reports that 23.3% of the dockworkers addressed were unable to work during the last three months due to muscular pain and 45% reported back pain in the last three months.

Casual dockworkers also reported mild discomforts such as pain, cramps and numbness (scores ≤ 5) when asked about anatomical regions where low back pain and dorsalgia occur. Despite mild discomfort, 27.6% of the workers who experience low back pain and 20.3% of those who presented dorsalgia reported the use of analgesic medications for musculoskeletal pain. The fact they use medications to minimize pain suggests pain is actually greater than the scores the workers assigned to pain. Additionally, a fear of showing frailty in the workplace by assigning high scores to pain, cramps or numbness, may explain the low scores assigned by the dockworkers to such discomfort in this study.

In this sense, it is believed that interventions intended to decrease physiological risks can minimize MSD among dockworkers and other workers exposed to such risks. One literature review addressing physiological risk factors and their relationship to occupational MSD in a computerized environment reports that working in a hectic environment may lead to high levels of muscular tension and strain, contributing to the development of MSD [37]. Even though port work is not totally computerized, the environment is tense due to an excess of hazards to which workers are exposed. Therefore, making the working environment at ports safer may help to prevent MSD. Additionally, this study collected self-reported data to identify occupational risks, which constitutes a limitation. More studies addressing occupational risks in the port environment are needed to quantify the occupational risks to which workers are exposed.

Literature reviews with the objective to establish a causal relationship between occupational tasks (flexion, torsion) [38] and occupational positions [39] among different types of workers report that it is unlikely that these are independent causes of low back pain. Therefore, more studies addressing casual dockworkers and workers in other professions are needed to identify the most prevalent causes in order to implement preventive measures for MSD; the intensification of MSD preventive programs is recommended by the WHO [7].

## 5. Conclusions

Low back pain and dorsalgia were prevalent in most of the casual dockworkers addressed in this study. The associated factor that remained in the regression model and therefore, contributed to increased prevalence of the two outcomes, was physiological occupational risk. Significant differences were found between association of other factors for each of the outcomes, such as: association between workload with low back pain, that is, total effort required and occupational frustration; and association between illegal drugs and being a quayside worker with dorsalgia. Even though quayside workers were those who most frequently presented inappropriate positions at work, given the nature of their work, we tend to claim that dorsalgia is directly related to this profession, which is corroborated by the regression model.

Additionally, note that dockworkers who experienced low back pain and/or dorsalgia were more perceptive regarding the risks to which they are exposed in their working environment.

The fact that no diagnostic imaging tests were presented constitutes a limitation in this study. Nonetheless, the report of each of the interviewees was confirmed in their workplace, as they either confirmed or denied having a medical diagnosis obtained in the Occupational Health and Safety Service or in another healthcare service. This study has an evaluative potential both for the scientific community that focuses on these specific workers or those working under similar conditions and for the population working in ports and those managing the workforce in regional and local ports to develop, in partnership with universities, strategies to support safe and healthy conditions for the work performed in ports.

## Figures and Tables

**Table 1:** Sample’s characterization (n = 232).

Variables	n = 232
**Age (years)—mean ± SD ^1^**	48.7 ± 10.4
**Age—n (%)**	
<40 years old	50 (21.6)
40 to 59 years old	140 (60.3)
≥60 years old	42 (18.1)
**Race—n (%)**	
Caucasian	130 (56.0)
Afro-descendent	54 (23.3)
Mixed race	34 (14.7)
Indigenous	6 (2.6)
Asian descendent	8 (3.4)
**Marital status—n (%)**	
Single	49 (21.1)
Married	141 (60.8)
Widowed	7 (3.0)
Separated/Divorced	35 (15.1)
**Education—n (%)**	
Illiterate	3 (1.3)
Incomplete Primary School	67 (28.9)
Complete Primary School	35 (15.1)
Incomplete high school	22 (9.5)
Complete high school	86 (37.1)
Some college studies	10 (4.3)
Bachelor’s degree or higher	9 (3.9)
Smoker—n (%)	59 (25.4)
Alcoholic—n (%)	119 (51.3)
Illegal drug use—n (%)	29 (12.5)
Self-medication for musculoskeletal pain—n (%)	77 (33.2)

^1^ Standard Deviation.

**Table 2:** Work data (n = 232).

Variables	n = 232
**Function—n (%)**	
Quayside workers	136 (58.6)
Longshoremen	79 (34.1)
Cargo checkers	17 (7.3)
**Time working in the sector (years)—mean ± SD ^1^**	24.3 ± 11.4
**Monthly income (Real)—md ^2^ (P25 ^3^–P75 ^4^)**	1016.94 (749.32–1338.07)
**Work hours—mean ± SD**	7.2 ± 2.4
**Robust variable/(hours versus years)—Md (P25–P75)**	150 (120–210)
**Work shift—n (%)**	
Only day shift	32 (13.8)
Only night shift	16 (6.9)
Night/Day shifts	184 (79.3)
**Scores assigned to workload demanded by tasks performed in the port—mean ± SD**	
Mental demand	13.0 ± 5.1
Physical demand	14.3 ± 5.0
Temporal demand	11.5 ± 5.7
Performance	16.1 ± 4.5
Total effort	14.9 ± 5.1
Level of frustration	9.8 ± 6.4

^1^ Standard deviation; ^2^ Median; ^3^ Percentile 25; ^4^ Percentile 75.

**Table 3:** Association of variables with dorsalgia and low back pain.

Variables ^1^	w/Low Back Pain (n = 162)	w/o Low Back Pain (n = 70)	*p*	w/Dorsalgia (n = 118)	w/o Dorsalgia (n = 114)	*p*
**Age (years)**	47.8 ± 9.8	50.8 ± 11.5	0.061 ^4^	48.4 ± 9.8	49.0 ± 11.0	0.701 ^4^
**Age group**			0.126 ^3^			0.472 ^3^
<40 years old	35 (21.6)	15 (21.4)		25 (21.2)	25 (21.9)	
40 to 59 years old	103 (63.6)	37 (52.9)		75 (63.6)	65 (57.0)	
≥60 years old	24 (14.8)	18 (25.7)		18 (15.3)	24 (21.1)	
**Race**			0.380 ^3^			0.113 ^3^
Caucasian	93 (57.4)	37 (52.9)		75 (63.6)	55 (48.2)	
Afro-descendant	38 (23.5)	16 (22.9)		23 (19.5)	31 (27.2)	
Mixed	24 (14.8)	10 (14.3)		16 (13.6)	18 (15.8)	
Indigenous	2 (1.2)	4 (5.7)		1 (0.8)	5 (4.4)	
Asian-descendant	5 (3.1)	3 (4.3)		3 (2.5)	5 (4.4)	
**Marital status**			0.049 ^3^			0.937 ^3^
Single	32 (19.8)	17 (24.3)		24 (20.3)	25 (21.9)	
Married	105 (64.8)	36 (51.4)		72 (61.0)	69 (60.5)	
Widowed	2 (1.2)	5 (7.1) ^2^		3 (2.5)	4 (3.5)	
Separated/Divorced	23 (14.2)	12 (17.1)		19 (16.1)	16 (14.0)	
**Education**			0.557 ^3^			0.806 ^3^
Illiterate/Incomplete primary school	53 (32.7)	17 (24.3)		37 (31.4)	23 (28.9)	
Complete primary school	22 (13.6)	13 (18.6)		20 (16.9)	15 (13.2)	
Incomplete high school	15 (9.3)	7 (10.0)		9 (7.6)	13 (11.4)	
Complete high school	61 (37.7)	25 (35.7)		41 (34.7)	45 (39.5)	
Some college studies	5 (3.1)	5 (7.1)		6 (5.1)	4 (3.5)	
Bachelor’s degree or more	6 (3.7)	3 (4.3)		5 (4.2)	4 (3.5)	
**Smoker**	39 (24.1)	20 (28.6)	0.577 ^3^	33 (28.0)	26 (22.8)	0.452 ^3^
**Alcoholic **	87 (53.7)	32 (45.7)	0.330 ^3^	66 (55.9)	53 (46.5)	0.191 ^3^
**Illegal drug use**	20 (12.3)	9 (12.9)	1.000 ^3^	20 (16.9)	9 (7.9)	0.059 ^3^
**Self-medication for musculoskeletal pain—n (%)**	64 (27.6)	13 (5.6)	0.002 ^3^	47 (20.3)	30 (12.9)	0.029 ^3^
**Function**			0.095 ^3^			0.567 ^3^
Quayside workers	96 (59.3)	40 (57.1)		66 (55.9)	70 (61.4)	
Longshoremen	58 (35.8)	21 (30.0)		44 (37.3)	35 (30.7)	
Cargo checkers	8 (4.9)	9 (12.9)		8 (6.8)	9 (7.9)	
**Monthly income ($) **	1016.94 (749.32–1338.07)	1070.46 (734.60–1338.07)	0.987 ^5^	1070.46 (749.32–1338.07)	936.65 (749.32–1338.07)	0.415 ^5^
**Time working in the sector (years)**	23.3 ± 10.5	26.6 ± 13.0	0.063 ^4^	24.1 ± 11.0	24.4 ± 11.8	0.822 ^4^
**Working hours**	7.38 ± 2.59	6.68 ± 1.96	0.025 ^4^	7.37 ± 2.65	6.96 ± 2.18	0.196 ^4^
**Robust variable (hours × years)**	150 (120–204)	153 (120–217)	0.642 ^5^	150 (120–212)	144 (120–206)	0.689 ^5^
**Work shift**			0.944 ^3^			0.548 ^3^
Only day shift	21 (13.0)	11 (15.7)		18 (15.3)	14 (12.3)	
Only night shift	11 (6.8)	5 (7.1)		6 (5.1)	10 (8.8)	
Night/Day shifts	130 (80.3)	54 (77.2)		94 (79.7)	90 (79)	
**Scores assigned to workload**						
Mental Demand	13.3 ± 4.8	12.2 ± 5.8	0.149 ^4^	13.5 ± 4.6	12.4 ± 5.5	0.101 ^4^
Physical Demand	14.9 ± 4.8	12.9 ± 5.3	0.008 ^4^	14.3 ± 5.0	14.2 ± 5.1	0.866 ^4^
Temporal Demand	12.0 ± 5.5	10.2 ± 6.0	0.027 ^4^	12.2 ± 5.5	10.7 ± 5.8	0.051 ^4^
Performance	16.7 ± 3.9	14.8 ± 5.5	0.012 ^4^	15.7 ± 4.4	16.6 ± 4.6	0.170 ^4^
Total effort	15.8 ± 4.5	13.0 ± 6.0	0.001 ^4^	15.6 ± 4.7	14.3 ± 5.5	0.053 ^4^
Level of frustration	10.7 ± 6.5	7.7 ± 5.7	0.001 ^4^	10.5 ± 6.6	9.0 ± 6.2	0.086 ^4^

^1^ Variables described by mean ± standard deviation, median (percentile 25–75) or n (%); ^2 ^Statistically significant association according to adjusted residuals test at a 5% level of significance; ^3 ^Pearson’s chi-square test; ^4 ^Student’s *t* test; ^5 ^Mann-Whitney test.

**Table 4:** Rates assigned by casual dockworkers for pain, cramps and numbness in anatomical regions.

Anatomical Regions	Pain	Cramps	Numbness
	Mean (±SD ^1^)	Mean (±SD ^1^)	Mean (±SD ^1^)
**Neck**	1.71 ± 3.19	0.03 ± 0.45	0.06 ± 0.65
**Upper back**	1.36 ± 2.91	0.13 ± 1.00	0.09 ± 0.83
**Medium back**	1.42 ± 3.04	0.10 ± 0.87	0.09 ± 0.80
**Low back**	3.65 ± 3.89	0.11 ± 0.89	0.06 ± 0.74

^1^ Standard deviation.

**Table 5:** Occupational risks (n = 232).

	w/Low Back Pain (n = 162) n (%)	w/o Low Back Pain (n = 70) n (%)	*p*-Value ^1^	w/Dorsalgia (n = 118) n (%)	w/oDorsalgia (n = 114) n (%)	*p* ^1^
**Physical**	157 (67.7)	69 (29.7)	0.048	115 (49.6)	111 (47.8)	0.966
**Chemical**	154 (66.4)	70 (30.2)	0.058	113 (48.7)	111 (47.8)	0.028
**Biological**	137 (59.1)	61 (26.3)	0.611	103 (44.4)	95 (40.9)	0.010
**Physiological**	154 (66.4)	64 (27.6)	0.001	112 (48.3)	106 (45.7)	0.031
**Psychosocial**	146 (62.9)	57 (24.6)	0.019	105 (45.3)	98 (42.2)	0.487

^1^ Pearson’s chi-square.

**Table 6:** Poisson regression analysis to avoid factors independently associated with dorsalgia or low back pain.

Outcomes	PR ^1^ (95% CI) ^2^	*p*
**Low back pain**		
Physiological occupational risk	1.32 (1.08–1.60)	0.006
Scores assigned to workload		
Total effort	1.03 (1.01–1.05)	0.014
Frustration	1.01 (1.00–1.03)	0.020
**Dorsalgia**		
Use of illegal drugs	1.34 (1.04–1.73)	0.023
Being a longshoreman	1.39 (1.05–1.85)	0.021
Physiological occupational risk	1.30 (1.01–1.67)	0.040

^1^ Prevalence ratio; ^2^ CI 95% = 95% Confidence Interval.

**Table 7:** Frequency of observation concerning body positions of dockworkers during work.

Positions Assumed During Port Work ^1^	Quayside Workers		Longshoremen		Cargo Checkers	
	n	%	n	%	n	%
Static position	19	33.9	02	28.6	01	33.3
Awkward static position	34	60.7	04	57.1	00	0.0
Kneeling	01	1.8	01	14.3	00	0.0
Kneeling in an awkward position	15	26.8	02	28.6	00	0.0
Sitting	12	21.4	03	42.9	00	0.0
Sitting in an awkward position	05	8.9	02	28.6	02	66.7
Lifting heavy loads	03	5.4	00	0.0	00	0.0

^1^ Positions according to WHO classifications [6].
